# Possible correlation between *Mycobacterium abscessu*s subsp. *massiliense* spondylodiscitis and spinal anesthesia: Diagnostic and therapeutic challenges

**DOI:** 10.1590/0037-8682-0009-2026

**Published:** 2026-09-25

**Authors:** Henrique Soares Casaccio, Júlia Lustosa Martinelli, Pedro Augusto Simão Vasconcellos, Mariani de Lima Garcia, Lucas de Noronha Lima, Matheus Oliveira Povoa, Andressa Caroline Paranhos, Alessa de Andrade Santana, Julia Domingues Gatti, Nanci Michele Saita Santos, Aline Gonzalez Vigani, Elisa Teixeira Mendes, Lucieni de Oliveira Conterno, Raquel Silveira Bello Stucchi, Francisco Hideo Aoki, Márcia Teixeira Garcia, Mariângela Ribeiro Resende, Antonio Camargo Martins

**Affiliations:** 1 Universidade Estadual de Campinas, Faculdade de Ciências Médicas, Departamento de Clínica Médica, Subárea Infectologia, Campinas, SP, Brasil.; 2 Rede Brasileira de Pesquisa em Tuberculose (REDE-TB), Rio de Janeiro, RJ, Brasil.

**Keywords:** Mycobacterium abscessus, Spondylodiscitis, Healthcare-associated infection

## Abstract

Spinal infections caused by *Mycobacterium abscessus* are rare and difficult to treat. We report the first case of *M. abscessus* subsp. *massiliense* spondylodiscitis temporally associated with spinal anesthesia. A 57-year-old man developed back pain after undergoing spinal anesthesia for prostate surgery. Six months later, decompressive laminectomy was performed; results for bacterial and fungal culture and molecular testing for *Mycobacterium tuberculosis* were negative. Two years later, a mycobacterial culture of material from a paravertebral abscess revealed the presence of *M. abscessus*, later identified as subsp. *massiliense*. Treatment with imipenem, amikacin, tigecycline, clofazimine, and clarithromycin resulted in clinical and radiological improvement. This report highlights the diagnostic and therapeutic challenges of this case.

## INTRODUCTION

Spondylodiscitis is an infection of the vertebral bodies and intervertebral discs resulting from hematogenous spread or direct inoculation of microorganisms during invasive procedures or trauma. Nontuberculous mycobacteria (NTM) should be considered in culture-negative or treatment-refractory spondylodiscitis. The diagnosis of *M. abscessus* depends on mycobacterial culture and molecular testing; however, these methods may only be available at reference centers. Treating *M. abscessus* infection often requires combination therapies with intravenous agents, and may be associated with severe toxicity.

Only a few cases of spinal infections caused by *M. abscessus* have been reported, none of which have been associated with neuraxial anesthesia. Herein, we report a case of *M. abscessus* subsp. *massiliense* spondylodiscitis that was temporally associated with spinal anesthesia. To provide context, we searched the PubMed database to identify cases of spinal infection caused by the *M. abscessus* complex across settings. This study was approved by the UNICAMP Research Ethics Committee (approval number 92895225.3.0000.5404).

## CASE REPORT

A 57-year-old Brazilian man born in Bahia, Northeast Brazil, sought care in São Paulo, Southeast Brazil, for dorsal pain at T12-L2, which had recently worsened. The patient had spondylodiscitis of unknown etiology and received empirical therapy, but showed no improvement.

The clinical course had begun approximately 2 years earlier (Figure 1), when the patient developed lumbar pain after spinal anesthesia for transurethral prostate resection. The pain progressed, and spondylodiscitis at T11-T12 was diagnosed approximately 30 days after the procedure, without evidence of spinal cord compression or paravertebral abscesses. Neither surgery nor culture was performed, and empirical treatment with oxacillin, metronidazole, and ciprofloxacin was administered for 60 days. Despite minimal reduction in pain and imaging findings showing little improvement, conservative management with outpatient follow-up was selected.

**FIGURE 1:**
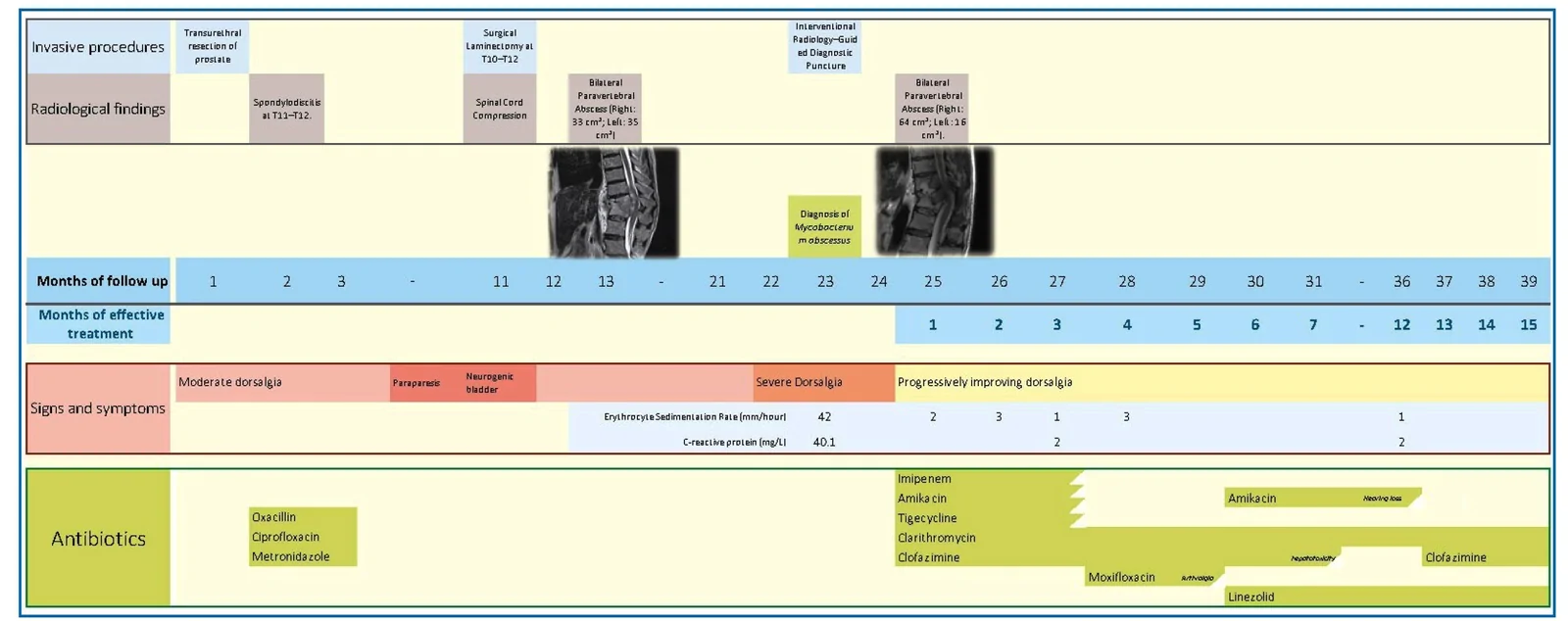
Patient’s clinical course.

Six months later, the dorsalgia worsened, and signs of spinal cord compression (bilateral lower limb paraparesis and neurogenic bladder) developed. Decompressive laminectomy from T10 to T12 was performed and a local biopsy specimen obtained. The results of the bacterial, mycobacterial, and fungal cultures and molecular testing for*M. tuberculosis* performed on the biopsy specimen were negative.

Despite improvement in our patient’s neurological symptoms, pain persisted. The patient missed follow-up for approximately 5 months. Because the pain worsened, he sought care at our quaternary referral center. At that time, he reported intense pain at T12-L2, but had no neurological signs. Throughout the 2-year course, he did not develop chronic cough, fever, night sweats, or weight loss. His medical history included a fall from height several years before undergoing prostate surgery. He had no history of previous surgical intervention or intravenous drug use.

The investigation revealed spondylodiscitis at T10-T11 and T11-T12, along with fractures of the T11 and T12 vertebral bodies. Marked foraminal stenosis was observed at T11-T12, compressing the nerve roots; anterior epidural soft tissue changes were also noted. Paravertebral collections, compatible with abscesses and extending into the right psoas muscle, were observed on the right (10 × 6.2 × 2 cm, 64.93 cm³) and left (4.6 × 1.8 × 3.8 cm, 16.47 cm³) sides. Laboratory findings were as follows: leukocyte count, 6.04 × 10⁹/L; C-reactive protein level, 40.1 mg/L; and erythrocyte sedimentation rate, 42 mm/h. The results of the serological tests for human immunodeficiency virus, hepatitis B, hepatitis C, and schistosomiasis were negative.

The paravertebral abscesses were drained using imaging guidance from interventional radiology, yielding 5 mL of purulent material for microbiological analysis. Mycobacterial culture test results identified *M. abscessus* 6 days after specimen collection. The results of routine bacterial and fungal culture tests and GeneXpert(Cepheid, Sunnyvale, CA, USA) testing for *M. tuberculosis*were negative.

Treatment was initiated according to the Brazilian protocol^1^ ^,^ ^2^, and comprised imipenem, amikacin, tigecycline, clofazimine, and clarithromycin. The patient remained hospitalized for 3 months. His outcomes were favorable, despite adverse effects that primarily included nausea, vomiting, and poor oral intake. No further spinal cord compression occurred; the patient’s vital signs remained stable.

No additional neurosurgical intervention was performed due to the patient’s clinical stability and the technical difficulties associated with accessing the affected site. Antimicrobial therapy was planned for at least 18 months. Three months after undergoing intravenous treatment, the patient was discharged, with oral clarithromycin, clofazimine, and moxifloxacin prescribed. Severe arthralgia attributed to moxifloxacin use required that the drug be replaced with amikacin and linezolid.

At month 7, clofazimine was discontinued because hepatotoxicity developed, and treatment continued with amikacin, linezolid, and clarithromycin. At month 13, amikacin was discontinued because hearing loss developed, and clofazimine was reintroduced with hepatic monitoring. The current regimen consists of clofazimine, linezolid, and clarithromycin.

The patient’s pain levels have progressively decreased since treatment initiation. Three-phase bone scintigraphy performed during the first month of treatment indicated marked bone remodeling at T11 and T12. At month 10, gallium-67 citrate imaging showed minimal tracer uptake at T11 and T12, suggesting reduced local infection and inflammation (Figure 2). The levels of inflammatory markers remained low.

**FIGURE 2:**
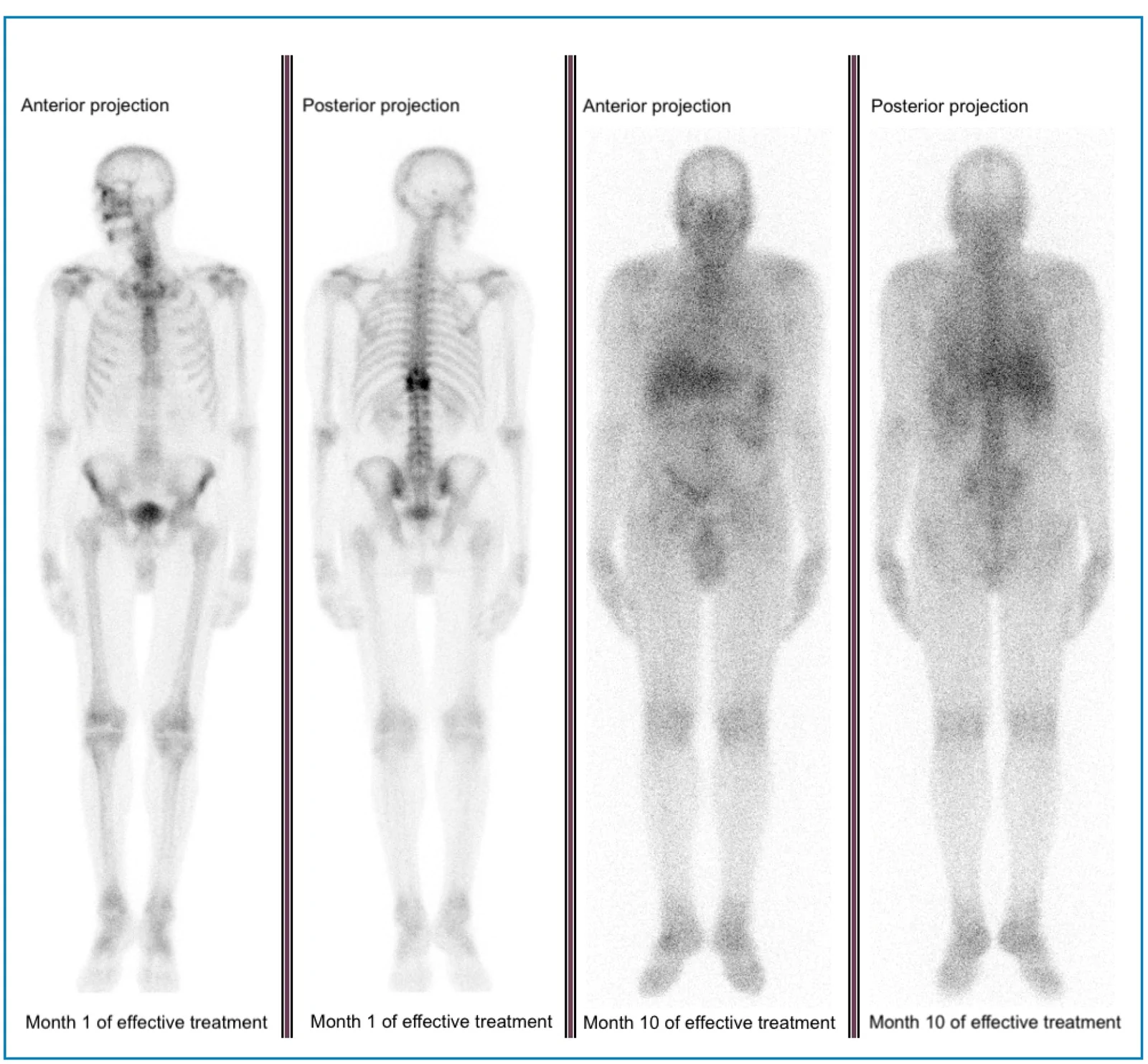
Longitudinal scintigraphic findings.

The isolate was sent to a mycobacterial reference laboratory at Instituto Adolfo Lutz for subspecies identification and antimicrobial susceptibility testing^3^ ^.^ At the end of month 3, laboratory test results confirmed the presence of *M. abscessus* subsp. *Massiliense*. Resistance to trimethoprim-sulfamethoxazole, ciprofloxacin, imipenem, moxifloxacin, cefoxitin, and tobramycin; susceptibility to amikacin and clarithromycin; and intermediate susceptibility to linezolid and doxycycline were also reported (Supplementary Material).

A limitation of this case was that spinal anesthesia and laminectomy were performed at outside institutions in another state, with initial follow-up occurring outside our center. Although the temporal relationship with spinal anesthesia suggested a procedure-associated event, the source of infection, any specific breach in aseptic technique or sterilization procedures, or water contamination could not be identified.

## DISCUSSION

Few cases of spondylodiscitis caused by NTM have been reported, although bone and joint infections caused by these organisms have been documented. In a French case series, 19.7% of NTM infections involved spondylodiscitis, 35.71% involved individuals with immunocompetence, and 64.29% involved patients with immunosuppression^4^. In a case series of Iranian patients with pyogenic spondylodiscitis, the causative pathogens were identified using molecular analysis in 36.84% of cases, and *M. abscessus*accounted for 23.8% of the cases^5^. This finding contrasts with those of other studies, in which *M. abscessus* was rarely identified.

*We identified 23 published cases of spinal infections caused by the M. abscessus complex,* including spondylodiscitis, vertebral osteomyelitis, and epidural or paravertebral abscesses. The therapeutic approaches and durations were heterogeneous (Table 1). In some cases, infection occurred in patients without immunosuppression, whereas five were related to the use of intravenous illicit drugs.

**TABLE 1:** Reported cases of *Mycobacterium abscessus* with spinal involvement.

Authors, year published	Sex, age	Immunosuppression	Underlying condition	Surgical intervention	Antimicrobial regimen	Treatment duration	Adverse effects	Outcome
Sarria et al., 1998	M, 53 y	No	IV drug use	Yes	1st: Amikacin, cefoxitin, clarithromycin; 2nd: Imipenem, cefoxitin, clarithromycin	12 months	Nephrotoxicity	Favorable
Chan et al., 2001	F, 16 y	No	Post-trauma	No	Clarithromycin	6 months	-	Favorable
Edwards et al., 2012	F, 65 y	No	Laminectomy (years prior)	No	Not initiated (lost to follow-up)	-	-	Lost to follow-up
Garcia et al., 2013	M, 44 y	No	IV drug use, hepatitis C	No	Cefoxitin, amikacin, clarithromycin	6 months	-	Favorable
Kato et al., 2014	F, 67 y	No	Hypothyroidism, hypertension	Yes	1st: Amikacin, clarithromycin, imipenem; 2nd: Clarithromycin	6 months	-	Favorable
Silva et al., 2015	F, 71 y	Yes	Heart transplant	No	1st: Clarithromycin, tigecycline; 2nd: Clarithromycin, linezolid	3 months	Hepatotoxicity	Favorable
Sheikh et al., 2017 (Case series - five cases)	-	-	Case 8: Addict Case 11: Diabetes mellitus, renal failure Case 17: Diabetes mellitus, metastatic lung cancer Case 41: Diabetes mellitus Case 48: -	-	-	-	-	-
Zhang et al., 2018	M, 51 y	Yes	SAPHO syndrome, glucocorticoid and methotrexate use	Yes	1st: Azithromycin, amikacin, linezolid, tigecycline; 2nd: Tigecycline, bedaquiline, clofazimine, cefoxitin	>15 months	QT prolongation, nausea, anorexia, malnutrition, peripheral neuropathy, myelosuppression	Favorable
Kadota et al., 2019	F, 63 y	No	-	No	1st: Imipenem, amikacin, clarithromycin; 2nd: Faropenem, levofloxacin, clarithromycin	24 months	-	Favorable
Moral et al., 2019	M, 82 y	No	-	Yes	Tigecycline, amikacin, meropenem; azithromycin, tigecycline, meropenem	-	Persistent nausea, onset of Barrett’s esophagus	Palliative
Shahi et al., 2020	F, 43 y	No	Post-invasive procedure (ozonotherapy)	Yes	1st: Amikacin, clarithromycin; 2nd: Clarithromycin	3 months	-	Favorable
Rahi et al., 2021	M, 55 y	Yes	IV drug use, hepatitis C, liver cirrhosis	No	1st: Meropenem, tigecycline, amikacin; 2nd: Amikacin, cefoxitin, imipenem	12 months	-	Favorable
Omori et al., 2022	M, 74 y	Yes	Glucocorticoids, diabetes mellitus, rheumatoid arthritis, lung cancer	Yes	1st: Clarithromycin, amikacin, imipenem; 2nd: Sitafloxacin, amikacin, imipenem	1.5 months	-	Death
Farid et al., 2023	M, 38 y	No	IV drug use, tattoo	Yes	1st: Amikacin, clarithromycin, imipenem; 2nd: Clarithromycin, imipenem; 3rd: Sitafloxacin, clarithromycin	18 months	Ototoxicity	Favorable
Bashti et al., 2023	M, 51 y	No	IV drug use, hepatitis C, arterial hypertension, hypercholesterolemia	Yes	1st: Levofloxacin, azithromycin, linezolid; 2nd: Amikacin, cefoxitin, clarithromycin; 3rd: Tigecycline, cefoxitin, clarithromycin	-	-	Favorable
Patel et al., 2024	F, 63 y	-	Failed prior treatment (no surgery)	Yes	Omadacycline, Linezolid, Imipenem	-	-	Favorable
Benjamin et al., 2024	M, 64 y	No	Diabetes mellitus, chronic kidney disease, arterial hypertension, cardiopathy	No	1st: Amikacin, imipenem, tigecycline, azithromycin, clofazimine; 2nd: Amikacin, imipenem, azithromycin, moxifloxacin, linezolid; 3rd: Amikacin, imipenem, azithromycin, moxifloxacin, clofazimine; 4th: Imipenem, azithromycin, moxifloxacin, clofazimine; 5th: Bedaquiline, azithromycin, moxifloxacin, clofazimine; 6th: Bedaquiline, azithromycin, imipenem, clofazimine; 7th: Imipenem, clofazimine, bedaquiline; 8th: clofazimine, tigecycline, imipenem, ceftazidime-avibactam	11 months	Ototoxicity, gastrotoxicity, hepatotoxicity, myelotoxicity, cardiotoxicity	Favorable
Bémer et al., 2024 (Case series- case 28)	-	No	-	No	1st: Clarithromycin, imipenem, amikacin; 2nd: Clarithromycin, tigecycline, ofloxacin; 3rd: Clarithromycin, ofloxacin, amikacin	11 months	-	Favorable
Bémer et al., 2024 (Case series- case 36)	-	No	-	Yes	-	-	-	Favorable

**F:** female; **M:** male; **IV:** intravenous. The full references for all studies cited in this table are provided in the Supplementary Material.

*M. abscessus*is among the three most prevalent NTM species worldwide, and infection is difficult to treat^6^ ^,^ ^7^. In a meta-analysis of *M. abscessus* pulmonary disease, the overall treatment success was 45.6%, with lower success reported for subsp. *abscessus* than for subsp. *massiliense* ^8^. The *M. abscessus* complex has three subspecies: *abscessus*, *bolletii*, and *massiliense*. Subspecies identification is clinically important because subsp. *abscessus* and *bolletii* have a functional erm(41) gene that confers inducible macrolide resistance, whereas subsp. *massiliense*lacks this gene and is susceptible to macrolides^9^.

Because most studies focus on pulmonary diseases caused by *M. abscessus*, no specific guidelines exist for treating extrapulmonary infections; therefore, treatment regimens for extrapulmonary infections are extrapolated. These include an initial parenteral attack phase followed by a maintenance phase. Standard recommendations include combining one or two parenteral agents with at least two oral drugs, followed by maintenance with 2-3 oral agents^9^.

Macrolides are considered first-line treatment if macrolide susceptibility is confirmed^9^ ^-^ ^11^. In cases of macrolide resistance, a more aggressive four-drug regimen is recommended^9^. Amikacin is considered the second most important agent^9^. Beyond macrolides and amikacin, no consensus exists regarding the optimal companion drugs, and treatment protocols include various agents, such as carbapenems, linezolid, tigecycline, fluoroquinolones, trimethoprim-sulfamethoxazole, bedaquiline, clofazimine, cefoxitin, and doxycycline^2^ ^,^ ^9^ ^,^ ^10^. Therefore, the choice of additional agents should be guided by susceptibility testing.

The combination of β-lactam antibiotics with synergistic activity has emerged as a potential treatment strategy for highly resistant infections^12^. However, current evidence remains insufficient to determine which combinations improve clinical outcomes. No consensus on optimal treatment duration has been reached. Some protocols define treatment duration based on culture conversion, whereas others recommend extended treatment when culture conversion cannot be confirmed^1^ ^,^ ^2^ ^,^ ^11^. Kwak et al. (2019) reported that total treatment duration was longer among patients with treatment failure than among those with treatment success^8^, underscoring the difficulty of defining optimal regimens and durations.

Treatment of *M. abscessus* infection often requires regimen modifications to reduce adverse effects, limiting standardization and requiring individualized management. In the present case, the patient developed joint, hepatic, and auditory toxicities during therapy.

The apparent increase in NTM infections may reflect the increasing use of invasive procedures, a growing population with immunosuppression, and improved laboratory detection. However, NTM infection should be viewed as more than a diagnostic or therapeutic challenge: It also raises concerns about healthcare-associated transmission (Supplementary Material).

Even when an NTM infection is suspected, etiological diagnosis may be delayed because mycobacterial culture requires specialized media and laboratory conditions. Furthermore, subspecies identification, molecular marker analysis, and antimicrobial susceptibility testing often require specialized laboratories. These constraints delay etiological diagnosis and contribute to poor outcomes, particularly in low- and middle-income settings, in which awareness and resources are limited.

Therefore, clinicians should maintain a high index of suspicion for NTM** **in patients with culture-negative or treatment-refractory spondylodiscitis, and ensure that tissue or fluid specimens are obtained from the infected site for microbiologic analysis. Healthcare services should also adopt validated reprocessing protocols, water quality control measures, and infection prevention practices.

## Data Availability

Data usage not reported, research data not used.
