# Supplementary material for: Possible correlation between Mycobacterium abscessus subsp. massiliense spondylodiscitis and spinal anesthesia: Diagnostic and therapeutic challenges
Source: Rev Soc Bras Med Trop. 2026 Sep 25;59:e0009-2026. doi: 10.1590/0037-8682-0009-2026 (PMC13619153; doi:10.1590/0037-8682-0009-2026)
Supplement: Supplementary file 1 [file 1678-9849-rsbmt-59-e0009-2026-md1.pdf]

Revista da Sociedade Brasileira de Medicina Tropical
Journal of the Brazilian Society of Tropical Medicine

Supplementary material

Microbiological identification and antimicrobial susceptibility testing

The *M. abscessus* complex was identified using the GenoType Mycobacterium CM assay (Hain Life Science GmbH, Nehren, Germany). Subspecies identification and molecular analysis of resistance-associated markers were performed using the GenoType NTM-DR assay (Hain Lifescience GmbH, Nehren, Germany), which differentiates between subspecies *abscessus*, *massiliense*, and *bolletii* and evaluates alterations in the *erm(41)* and *rrl* genes, which are associated with macrolide resistance, and in *rrs*, which is associated with aminoglycoside resistance. The isolate was identified as *M. abscessus* subsp. *massiliense*. PCR identified a characteristic 274-bp deletion in *erm(41)*, resulting in a truncated, nonfunctional gene and confirming the subspecies identification[1].

Minimum inhibitory concentrations (MICs) were determined by broth microdilution according to the Clinical and Laboratory Standards Institute guidelines[2]. For clarithromycin testing was extended to 14 days to assess inducible resistance[3]. The isolate was susceptible to clarithromycin (MIC, 0.5 µg/mL), consistent with the molecular profile of *M. abscessus* subsp. *massiliense*.

Published cases of *Mycobacterium abscessus* spondylodiscitis by country

Authors	Country where the case was reported	Year published
Sarria et al.[4]	United States	1998
Chan et al.[5]	United States	2001
Edwards et al.[6]	United StatesH	2012
Garcia et al.[7]	United States	2013
Kato et al.[8]	Japan	2014
Silva et al.[9]	Spain	2015
Sheikh et al.[10]	Iran	2017
Zhang et al.[11]	Australia	2018
Kadota et al.[12]	Japan	2019
Moral et al.[13]	United States	2019
Shahi et al.[14]	India	2020
Rahi et all.[15]	United States	2021
Omori et al.[16]	Japan	2022
Farid et al.[17]	Japan	2023
Bashti et al.[18]	United States	2023
Patel et al.[19]	United States	2024
Benjamin et al.[20]	United Kingdom	2024
Bémer et al.[21]	France	2024

Healthcare-associated outbreaks of *Mycobacterium abscessus* in Brazil

Since 2004, several Brazilian states experienced large outbreaks of skin and soft tissue infections caused by rapidly growing mycobacteria, mainly associated with surgical procedures. A genomic epidemiological study demonstrated that these outbreaks were driven by clonal dissemination of *Mycobacterium abscessus* subsp. *massiliense* and identified a lineage with marked persistence in healthcare environments and evidence of adaptation to surgical care settings[22]. Some of these outbreaks were associated with the limited efficacy of glutaraldehyde-based high-level disinfection against this pathogen, particularly during the reprocessing of reusable medical devices[23,24].

Beyond the surgical setting, healthcare-associated outbreaks and pseudo-outbreaks caused by NTM have also been linked to failures in the reprocessing of bronchoscopes, endoscopes, and other medical devices, including contamination of cleaning and disinfection water[25,26]. These vulnerabilities may reflect operational failures, gaps in technical training, and limited adherence to validated reprocessing protocols[27].

Hospital water systems are a potential source of transmission. Exposure may occur through tap water utilization in patient care[28]; aerosols generated by water-based heating or cooling devices, even without direct contact with the infected site[29], or direct inoculation from contaminated medical products, solutions, or supplies[30]. Aesthetic procedures and surgical tourism constitute additional risk settings, particularly when performed in contexts with variable infection prevention standards and inconsistent material reprocessing practices[31,32].

References for the Supplementary Material

1. Carvalho NFG de, Pavan F, Sato DN, Leite CQF, Arbeit RD, Chimara E. Genetic correlates of clarithromycin susceptibility among isolates of the Mycobacterium abscessus group and the potential clinical applicability of a PCR-based analysis of erm(41). J Antimicrob Chemother. 2018;73(4):862-6.
2. Clinical and Laboratory Standards Institute (CLSI). Susceptibility testing of mycobacteria, nocardiae, and other aerobic actinomycetes; approved standard. 2nd ed. CLSI document M24-A2. Wayne (PA): CLSI; 2011. 61 p.

3. Garcia de Carvalho NF, Sato DN, Pavan FR, Ferrazoli L, Chimara E. Resazurin microtiter assay for clarithromycin susceptibility testing of clinical isolates of Mycobacterium abscessus group. J Clin Lab Anal. 2016;30(5):751-5.
4. Sarria JC, Chutkan NB, Figueroa JE, Hull A. Atypical mycobacterial vertebral osteomyelitis: case report and review. Clin Infect Dis. 1998;26(2):503-5.
5. Chan ED, Kong PM, Fennelly K, Dwyer AP, Iseman MD. Vertebral osteomyelitis due to infection with nontuberculous Mycobacterium species after blunt trauma to the back: 3 examples of the principle of locus minoris resistentiae. Clin Infect Dis. 2001;32(10):1506-10.
6. Edwards C, Diveronica M, Abel E. Epidural abscess caused by Mycobacterium abscessus. Am J Case Rep. 2012;13:180-2.
7. Garcia DC, Sandoval-Sus J, Razzaq K, Young L. Vertebral osteomyelitis caused by Mycobacterium abscessus. BMJ Case Rep. 2013;2013:bcr2013009597.
8. Kato S, Murakami H, Demura S, Yoshioka K, Hayashi H, Yokogawa N, et al. Vertebral osteomyelitis caused by Mycobacterium abscessus surgically treated using antibacterial iodine-supported instrumentation. Case Rep Orthop. 2014;2014:197061.
9. Silva JT, López-Medrano F, Fernández-Ruiz M, San-Juan R, Ruiz-Cano MJ, Delgado JF, et al. Mycobacterium abscessus pulmonary infection complicated with vertebral osteomyelitis in a heart transplant recipient: case report and literature review. Transpl Infect Dis. 2015;17(3):418-23.

10. Sheikh AF, Khosravi AD, Goodarzi H, Nashibi R, Teimouri A, Motamedfar A, et al. Pathogen identification in suspected cases of pyogenic spondylodiscitis. Front Cell Infect Microbiol. 2017;7:60.
11. Zhang Z, Su WY, Owler PBK. Disseminated Mycobacterium abscessus infection with spondylodiscitis of thoracic spine. J Surg Case Rep. 2018;2018(6):rjy141.
12. Kadota N, Shinohara T, Hino H, Goda Y, Murase Y, Mitarai S, et al. Mycobacterium abscessus subsp. abscessus infection progressing to empyema from vertebral osteomyelitis in an immunocompetent patient without pulmonary disease: a case report. BMC Pulm Med. 2019;19(1):100.
13. Moral MZ, Desai K, Arain AR, O'Leary RE, Haddad SF, Lawrence JP. Mycobacterium abscessus-associated vertebral osteomyelitis in an immunocompetent patient: a rare case report and literature review. Spinal Cord Ser Cases. 2019;5(1):53.
14. Shahi PB, Panigrahi V, Adsul N, Kumar M, Acharya S, Kalra KL, et al. Mycobacterium abscessus mimicking tubercular spondylodiscitis following ozone therapy: a case report and review of literature. Surg Neurol Int. 2020;11:63.
15. Rahi MS, Reyes SP, Parekh J, Gunasekaran K, Amoah K, Rudolph D. Disseminated Mycobacterium abscessus infection and native valve endocarditis. Respir Med Case Rep. 2021;32:101331.
16. Omori K, Kitagawa H, Tadera K, Naka Y, Sakamoto S, Kamei N, et al. Vertebral osteomyelitis caused by Mycobacteroides abscessus subsp.

abscessus resulting in spinal cord injury due to vertebral body fractures. J Infect Chemother. 2022;28(2):290-4.

17. Farid F, Nakamae T, Kamei N, Omori K, Maruyama T, Nakao K, et al. Vertebral osteomyelitis due to Mycobacterium abscessus subsp. massiliense with paravertebral abscess: a case report and review. J Infect Chemother. 2023;29(9):922-6.
18. Bashti M, Kumar V, Cote I, Peterson EC, Basil GW. Recurrent osteomyelitis caused by Mycobacterium abscessus necessitating surgical decompression and revision surgery with interbody fusion. Cureus. 2023;15(1):e33668.
19. Patel J, Malkoc A, Ghauri MS, Amin L, Petersen M, Cochrane J, et al. Anterior and posterior surgical approach for vertebral lumbar Mycobacterium abscessus osteomyelitis. J Surg Case Rep. 2024;2024(8):rjae521.
20. Benjamin A, Stevenson DR, Melzer M. Disseminated Mycobacterium abscessus with bone and central nervous system (CNS) involvement - a case report and review of the literature. Clin Infect Pract. 2024;23:100372.
21. Bémer P, Aubry A, Schramm F, Koebel C, Revillet H, Baltes V, et al. Clinical features and treatment outcomes of bone and joint nontuberculous mycobacterial infections according to immune status: a 9-year retrospective observational cohort. Int J Infect Dis. 2024;146:107122.
22. Everall I, Nogueira CL, Bryant JM, Sánchez-Busó L, Chimara E, Duarte R da S, et al. Genomic epidemiology of a national outbreak of post-surgical

Mycobacterium abscessus wound infections in Brazil. Microb Genom. 2017;3(5):e000111.

23. Lorena NS de O, Pitombo MB, Côrtes PB, Maya MCA, Silva MG da, Carvalho AC da S, et al. Mycobacterium massiliense BRA100 strain recovered from postsurgical infections: resistance to high concentrations of glutaraldehyde and alternative solutions for high level disinfection. Acta Cir Bras. 2010;25(5):455-9.
24. Monego F, Duarte RS, Nakatani SM, Araújo WN, Riediger IN, Brockelt S, et al. Molecular identification and typing of Mycobacterium massiliense isolated from postsurgical infections in Brazil. Braz J Infect Dis. 2011;15(5):436-41.
25. Fernandes Garcia de Carvalho N, Rodrigues Mestrinari AC, Brandão A, Souza Jorge L, Franco C, da Silveira Paro Pedro H, et al. Hospital bronchoscopy-related pseudo-outbreak caused by a circulating Mycobacterium abscessus subsp. massiliense. J Hosp Infect. 2018;100(3):e138-41.
26. Guimarães T, Chimara E, do Prado GVB, Ferrazoli L, Carvalho NGF, Simeão FC dos S, et al. Pseudooutbreak of rapidly growing mycobacteria due to Mycobacterium abscessus subsp bolletii in a digestive and respiratory endoscopy unit caused by the same clone as that of a countrywide outbreak. Am J Infect Control. 2016;44(11):e221-6.
27. von Glehn MP, Salcedo G, Batista TGS, Caballero LER, Frank CHM, Cunha EAT, et al. Outbreak of infections by Mycobacterium abscessus subsp. abscessus associated with cosmetic surgeries in the Brazil/Paraguay

border: a binational cohort study, 2021-2022. Epidemiol Serv Saude. 2025;34:e20250516.

28. Baker AW, Lewis SS, Alexander BD, Chen LF, Wallace RJ, Brown-Elliott BA, et al. Two-phase hospital-associated outbreak of Mycobacterium abscessus: investigation and mitigation. Clin Infect Dis. 2017;64(7):902-11.
29. Seidelman JL, Baker AW, Lewis SS, Warren BG, Barrett A, Graves A, et al. A cluster of three extrapulmonary Mycobacterium abscessus infections linked to well-maintained water-based heater-cooler devices. Infect Control Hosp Epidemiol. 2024;45(5):644-50.
30. Torres-Coy JA, Rodríguez-Castillo BA, Pérez-Alfonzo R, de Waard JH. Source investigation of two outbreaks of skin and soft tissue infection by Mycobacterium abscessus subsp. abscessus in Venezuela. Epidemiol Infect. 2016;144(5):1117-20.
31. Furuya EY, Paez A, Srinivasan A, Cooksey R, Augenbraun M, Baron M, et al. Outbreak of Mycobacterium abscessus wound infections among “lipotourists” from the United States who underwent abdominoplasty in the Dominican Republic. Clin Infect Dis. 2008;46(8):1181-8.
32. Newman MI, Camberos AE, Ascherman J. Mycobacteria abscessus outbreak in US patients linked to offshore surgicenter. Ann Plast Surg. 2005;55(1):107-10.
